# Structural Basis for Recognition of Human Enterovirus 71 by a Bivalent Broadly Neutralizing Monoclonal Antibody

**DOI:** 10.1371/journal.ppat.1005454

**Published:** 2016-03-03

**Authors:** Xiaohua Ye, Chen Fan, Zhiqiang Ku, Teng Zuo, Liangliang Kong, Chao Zhang, Jinping Shi, Qingwei Liu, Tan Chen, Yingyi Zhang, Wen Jiang, Linqi Zhang, Zhong Huang, Yao Cong

**Affiliations:** 1 Vaccine Research Center, Key Laboratory of Molecular Virology & Immunology, Institut Pasteur of Shanghai, Shanghai Institutes for Biological Sciences, Chinese Academy of Sciences, Shanghai, China; 2 National Center for Protein Science Shanghai, State Key Laboratory of Molecular Biology, Institute of Biochemistry and Cell Biology, Shanghai Institutes for Biological Sciences, Chinese Academy of Sciences, Shanghai, China; 3 Comprehensive AIDS Research Center, Collaborative Innovation Center for Diagnosis and Treatment of Infectious Diseases, School of Medicine, Tsinghua University, Beijing, China; 4 Shanghai Science Research Center, Chinese Academy of Sciences, Shanghai, China; 5 Markey Center for Structural Biology, Department of Biological Sciences, Purdue University, West Lafayette, Indiana, United States of America; University of North Carolina at Chapel Hill, UNITED STATES

## Abstract

Enterovirus 71 (EV71) is the main pathogen responsible for hand, foot and mouth disease with severe neurological complications and even death in young children. We have recently identified a highly potent anti-EV71 neutralizing monoclonal antibody, termed D5. Here we investigated the structural basis for recognition of EV71 by the antibody D5. Four three-dimensional structures of EV71 particles in complex with IgG or Fab of D5 were reconstructed by cryo-electron microscopy (cryo-EM) single particle analysis all at subnanometer resolutions. The most critical EV71 mature virion-Fab structure was resolved to a resolution of 4.8 Å, which is rare in cryo-EM studies of virus-antibody complex so far. The structures reveal a bivalent binding pattern of D5 antibody across the icosahedral 2-fold axis on mature virion, suggesting that D5 binding may rigidify virions to prevent their conformational changes required for subsequent RNA release. Moreover, we also identified that the complementary determining region 3 (CDR3) of D5 heavy chain directly interacts with the extremely conserved VP1 GH-loop of EV71, which was validated by biochemical and virological assays. We further showed that D5 is indeed able to neutralize a variety of EV71 genotypes and strains. Moreover, D5 could potently confer protection in a mouse model of EV71 infection. Since the conserved VP1 GH-loop is involved in EV71 binding with its uncoating receptor, the scavenger receptor class B, member 2 (SCARB2), the broadly neutralizing ability of D5 might attribute to its inhibition of EV71 from binding SCARB2. Altogether, our results elucidate the structural basis for the binding and neutralization of EV71 by the broadly neutralizing antibody D5, thereby enhancing our understanding of antibody-based protection against EV71 infection.

## Author Summary

Hand, foot and mouth disease (HFMD) caused by enterovirus infection is an infectious disease affecting millions of young children annually. Enterovirus 71 (EV71) is the major causative agent of severe HFMD with central nervous system complications. However, no prophylactic vaccine or therapeutic drug is available against EV71 so far. We previously identified a murine monoclonal antibody D5 with potent neutralization effect on EV71, yet its working mechanism remains elusive. In the current study we aim to unravel the structural basis of D5-mediated neutralization of EV71. Relative high-resolution cryo-EM analysis of EV71 particles in complex with IgG or Fab of D5 revealed a bivalent binding mode of D5 across the 2-fold axis of EV71 virion. We also found that the CDR3 of D5 heavy chain bound the VP1 GH-loop of EV71, which represents a broadly neutralizing epitope and is thought to mediate EV71 binding with its uncoating receptor SCARB2. Based on these observations, we propose that D5 neutralizes EV71 infection through competing with SCARB2 for a binding site at the VP1 GH-loop and/or inhibiting conformational change of the virus. We further showed that D5 treatment efficiently protected mice from lethal EV71 infection. Our work provides information that may facilitate the development of D5 antibody-derived anti-EV71 drugs.

## Introduction

Enterovirus 71 (EV71) is a member of the *enterovirus* genus of the *picornaviradae* family. EV71 infection may cause severe hand, foot and mouth disease (HFMD) associated with neurological complications, such as encephalitis, neurogenic pulmonary edema, and even death in children under 6 years old [1–3].

Cell cultures-derived EV71 exists in two icosahedral particle forms, one is the non-infectious empty particle (termed E-particle or procapsid) consisting of 60 copies of VP0, VP1 and VP3 proteins but lacking the viral genome, and the other is the infectious mature virion (termed F-particle) bearing the RNA genome and VP1, VP3, VP2 and VP4 proteins (the latter two result from cleavage of VP0) [4–6]. There is a surface depression called “canyon” around the 5-fold-related plateaus of both E- and F-particles [5,6]. Viral receptors bind the canyons of some enteroviruses such as poliovirus to trigger virus uncoating during infection [7]. Several molecules have been identified as the cellular receptors for EV71 [8], such as the scavenger receptor class B member 2 (SCARB-2) [9], p-selectin glycoprotein ligand-1 (PSGL-1) [10] and heparin sulfate glycosaminoglycan [11]. However, the receptor binding sites on EV71 virions have not been determined by structural analysis.

Passive transfer of neutralizing antisera from mice immunized with inactivated EV71 virus or recombinant virus-like particles (VLPs) has been shown to protect recipient mice against lethal challenge [12–15], demonstrating that antibodies are major component in EV71 immunity. These results also imply the potential of developing neutralizing antibody-based drugs for prevention and treatment of EV71 infections. Several neutralizing monoclonal antibodies (MAbs) against EV71 have been generated and tested in mouse models [16,17], but their potencies were only modest and their breadths of neutralization have not been determined. Very few studies have been conducted to unravel the mechanisms underlying MAb-mediated EV71 neutralization. Recently, it was shown that the MAb termed E18 could neutralize EV71 infection by inducing release of the viral RNA genome [18]. In previous studies, cryo-EM structures of EV71 in complex with antibody were resolved to resolutions of 16 Å and 8.8 Å [18,19]. In general, the mechanisms for EV71 neutralization by MAbs remain largely unknown, partly due to the lack of high-resolution structural information.

Previously, we obtained three neutralizing MAbs (D5, H7 and C4) from mice immunized with recombinant EV71 VLP [20]. Among them, MAb D5 showed the most potent neutralizing activity against EV71 [20]. But the underlying structural mechanism of EV71 neutralization by D5 antibody has remained elusive. In the current study, we presented four subnanometer-resolution cryo-EM structures of EV71 F-particle, E-particle and VLP in complex with D5 intact IgG or its antigen-binding fragment (Fab), with the most critical F-particle-Fab structure resolved to 4.8 Å resolution, which represents a notable improvement in cryo-EM studies of virus-Fab/antibody complexes. Through a combination of structural, virological and biochemical analyses, we explored the binding interface between D5 and EV71 to define the D5 epitope. Our results reveal a unique bivalent binding pattern of D5 on the enterovirus EV71, which may neutralize EV71 infectivity by blocking its receptor binding and/or by hindering its ability to undergo conformational changes required for release of its genetic material. We further demonstrated that D5 can efficiently confer *in vivo* protection against EV71 infection.

## Results

### Cryo-EM reconstructions of D5-bound EV71 particles

**Fig 1:**
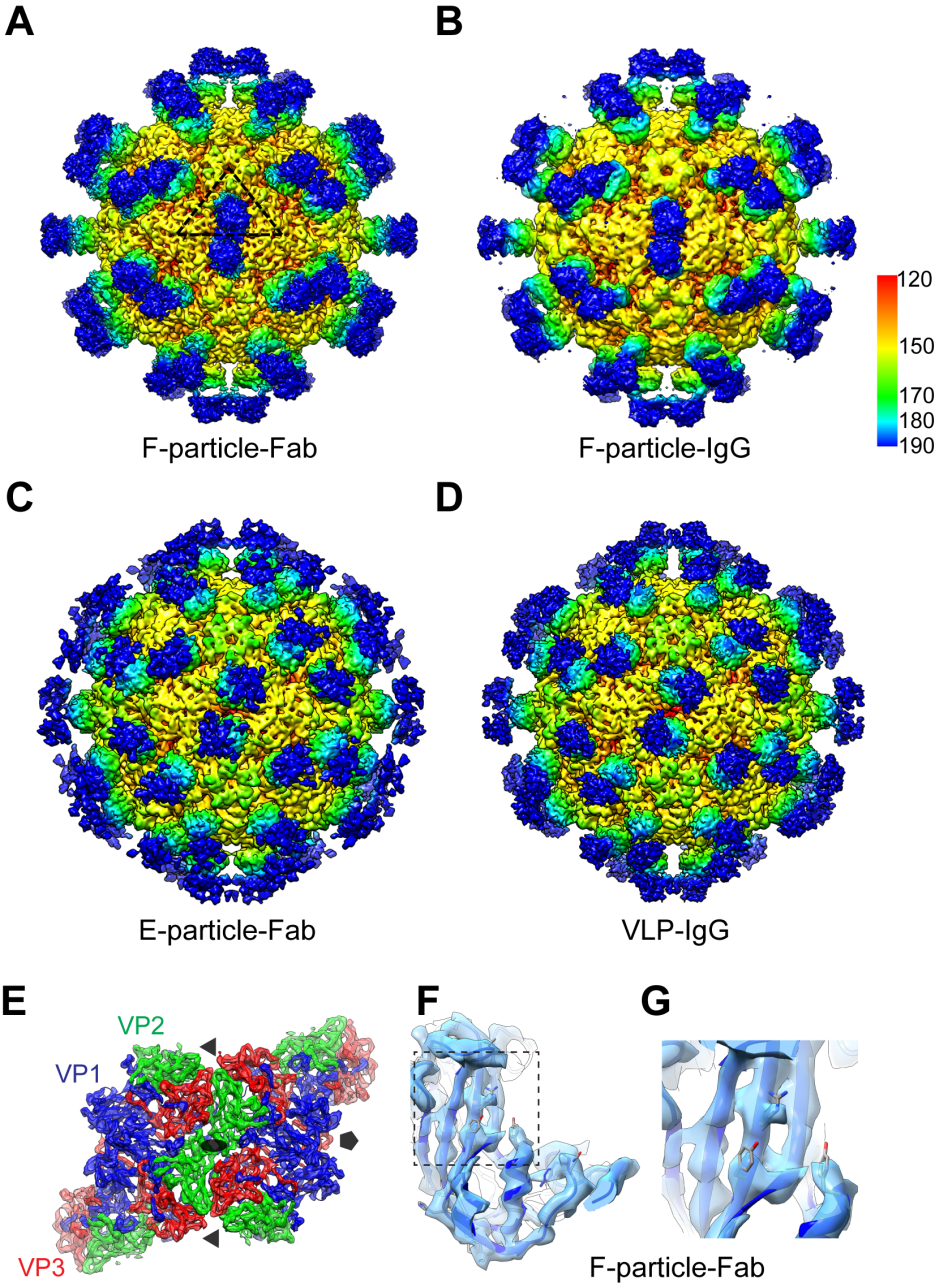
Cryo-EM maps of different EV71 particles or VLP in complex with D5 Fab or intact IgG. **(A)** F-particle in complex with Fab. One icosahedral asymmetric unit of the capsid is indicated by a black triangle. **(B)** F-particle in complex with IgG. The color bar labels the corresponding radius from the center of the sphere (unit in Å). **(C)** E-particle in complex with Fab. **(D)** VLP in complex with IgG. The Fab components of the complexes are rendered in green to blue color. In the IgG bound complexes (F-particle-IgG and VLP-IgG), the density of the Fc region of the antibody could not be resolved owning to its extremely dynamic nature. **(E)** F-particle-Fab map with fitted models of six adjacent protomers around the 2-fold axis. The Fab densities were removed. VP1, VP2 (VP0) and VP3 structures and densities are shown in blue, green and red, respectively. Same color schema was followed throughout. Positions of the 2-fold, 3-fold and 5-fold icosahedral symmetry axes are indicated as grey oval, triangles, and pentagons, respectively. **(F)** The segmented density of the VP1 compact region from the F-particle-Fab map with the fitted model. **(G)** Expanded view of a representative portion of the map and model displayed in Fig 1F.

To thoroughly understand the structural mechanism of how D5 binds EV71 and inhibits its infection, we determined four cryo-EM structures of D5 full IgG or its Fab in complex with EV71 mature virion (F-particle), procapsid (E-particle) or VLP, i.e. F-particle-Fab, F-particle-IgG, E-particle-Fab and VLP-IgG (Fig 1A–1D and S1 Table). The reconstructions were carried out by utilizing *jspr* software package [21]. In the representative micrographs, small protrusions are readily visible on the surfaces of EV71 particles or VLP (S1 Fig), indicating the binding of D5 IgG or Fab.

The cryo-EM structures of the F-particle-Fab and E-particle-Fab complexes were reconstructed to resolutions of 4.8 Å and 6 Å, respectively (Fig 1A and 1C and S2 Fig). In the capsid of the F-particle-Fab map, most of the β-strands were observed to be separated and several bulky side chains could be visualized (Fig 1F and 1G), demonstrating the resolution at the range of 4.5–4.8 Å. This is further validated by our local resolution evaluation using *Resmap* [22], showing that the capsid resolution is 4.5 Å (S3 Fig). Previously, the virus-Fab cryo-EM structures have been limited to around 10 Å resolutions due to conformational and compositional heterogeneity [18,23,24], with only a few cases having reached 6–8.8 Å resolutions [25–29]. Moreover, to dissect the binding profile of the intact IgG of D5 with EV71, and to compare it with that of the Fab, we also reconstructed the structures of the F-particle-IgG and VLP-IgG complexes, which yielded overall resolutions of 7.2 Å and 5.5 Å, respectively (Fig 1B and 1D and S2 Fig).

Analyses of these four maps indicated very similar overall structures for the F-particle-IgG and F-particle-Fab complexes (S4A and S4B Fig). The correlation score between the two complete maps was calculated to be 0.981 (S2 Table). The structure of VLP-IgG was also observed to be very similar to that of E-particle-Fab (S4C and S4D Fig), with a correlation score of 0.984 between their maps (S2 Table). These results indicate that the presence of the Fc fragment did not induce significant structural alternations in the bound virus particle or VLP, which therefore validates the feasibility of using Fab-virus structure, usually at higher resolution, to investigate the structural mechanism of IgG binding to virus.

These structures demonstrate that D5 antibodies bind to the tips of the three-blade propeller-like features on the surface of EV71 particle, which is located adjacent to the canyon and near the 2-fold axis (Fig 1A and S4 Fig). Moreover, the location and geometry of bound IgG/Fab also suggest that all 60 equivalent positions on the EV71 surface may in principle be simultaneously occupied by D5 IgG/Fab. In our maps, the Fab density consists of two lobes bridged by a hollow middle region (marked by red arrow in the right panel of S4A and S4B Fig), which is a characteristic feature of the Fab structure [30]. Notably, the two-lobed D5 Fab appears to be bound nearly perpendicularly to the F-particle surface (highlighted by black arrow-heads in the left panel of S4A and S4B Fig), but tilted relative to the E-particle and VLP surfaces, with adjacent Fabs tilted in opposite directions (highlighted by black arrow-heads in S4C and S4D Fig). This difference in Fab orientation could result from conformational differences for the slightly expanded (~5% in diameter) E-particle and VLP compared with the F-particle [5].

### A bivalent binding mode of D5 on EV71

**Fig 2:**
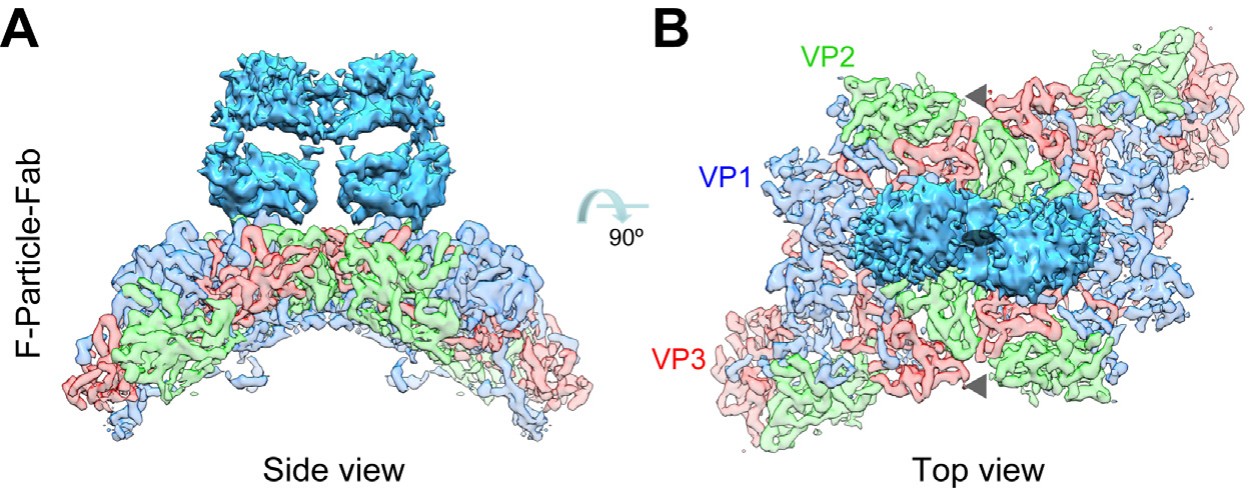
Close-up views of the antibody-virus junction. **(A)** The side and **(B)** top views of the cryo-EM density of the junction between the D5 Fab (deep sky blue) and the six adjacent EV71 protomers around the 2-fold axis. Similar color schema as in Fig 1E was adopted for the capsid densities but more transparent, and the Fab density was shown in deep sky blue.

In both the F-particle-Fab and F-particle-IgG maps, two adjacent Fabs appear to reside face-to-face across the 2-fold axis and their upper lobes are in contact (Fig 2), indicating a bivalent binding of intact D5 antibody on the EV71 F-particle. Intriguingly, this bivalent binding mode could cross-link adjacent protomers across the 2-fold axis, which may potentially prevent the viral capsid proteins from undergoing conformational changes required for viral genome release through the 2-fold channel [31]. In contrast, the adjacent Fabs in the E-particle-Fab and VLP-IgG are tilted further apart and their upper lobes are not in contact with each other (indicated by black arrow heads in S4C and S4D Fig).

In order to gather further evidence of D5 bivalent binding, we compared the neutralization potency of D5 IgG and its Fab fragment. By using a standard neutralization assay, the IC50 of D5 IgG was determined to be 2.23 nM, whereas that of Fab was 37.40 nM (S5A Fig), indicating that D5 IgG was 17 times more potent at neutralization than Fab in this assay. This demonstrates that D5 IgG is much more potent than its Fab in conferring neutralization, likely due to contribution of additional effects of the bivalent binding mode of intact IgG on F-particle.

To determine whether the increase in binding avidity plays an important role in the increased neutralization potency by IgG over Fab, we measured the avidities of D5 IgG and Fab for the EV71 F-particle by using a bio-layer interferometry (BLI) assay (S5C and S5D Fig). The KD values of D5 IgG and Fab were determined to be 6 nM and 15 nM, respectively, suggesting that the avidity of the D5 IgG only increased 2.5 times than that of the Fab. This slightly increased avidity alone is difficult to explain the obviously higher neutralizing ability (17 times) of D5 IgG over its Fab.

IgG is known to cause aggregation of virus particles. To test whether aggregation is the major cause of the increased neutralization potency by IgG over Fab, we carried out a post-attachment neutralization assay. In this assay, IgG or Fab was added after the virus had attached to target cells, thus eliminating the possibility of aggregation of virus particles mediated by intact IgG. We found that the IC50s of D5 IgG and its Fab fragment in this experiment were 10.12 nM and 191.4 nM, respectively (S5B Fig). These data indicate that, even without the contribution of antibody-mediated virus aggregation, neutralization by D5 IgG is still 19 times more potent than by its Fab fragment.

Altogether, the above results strongly suggest that effect of bivalent binding, other than increased binding avidity or inter-particle cross-linking, is mainly responsible for the significantly stronger neutralization potency of IgG over Fab.

### Footprints of D5 on EV71 particles

We produced partial pseudo-atomic models of the complexes by fitting the crystal structures of the EV71 F-particle (PDB ID: 3VBS) and E-particle (PDB ID: 3VBU) [5] into the corresponding cryo-EM density maps. The crystal structures fit well into the cryo-EM densities of the capsids (Fig 1E–1G and S2B Fig), suggesting that neither D5 Fab binding nor D5 antibody binding induced a significant conformational change in the capsid.

**Fig 3:**
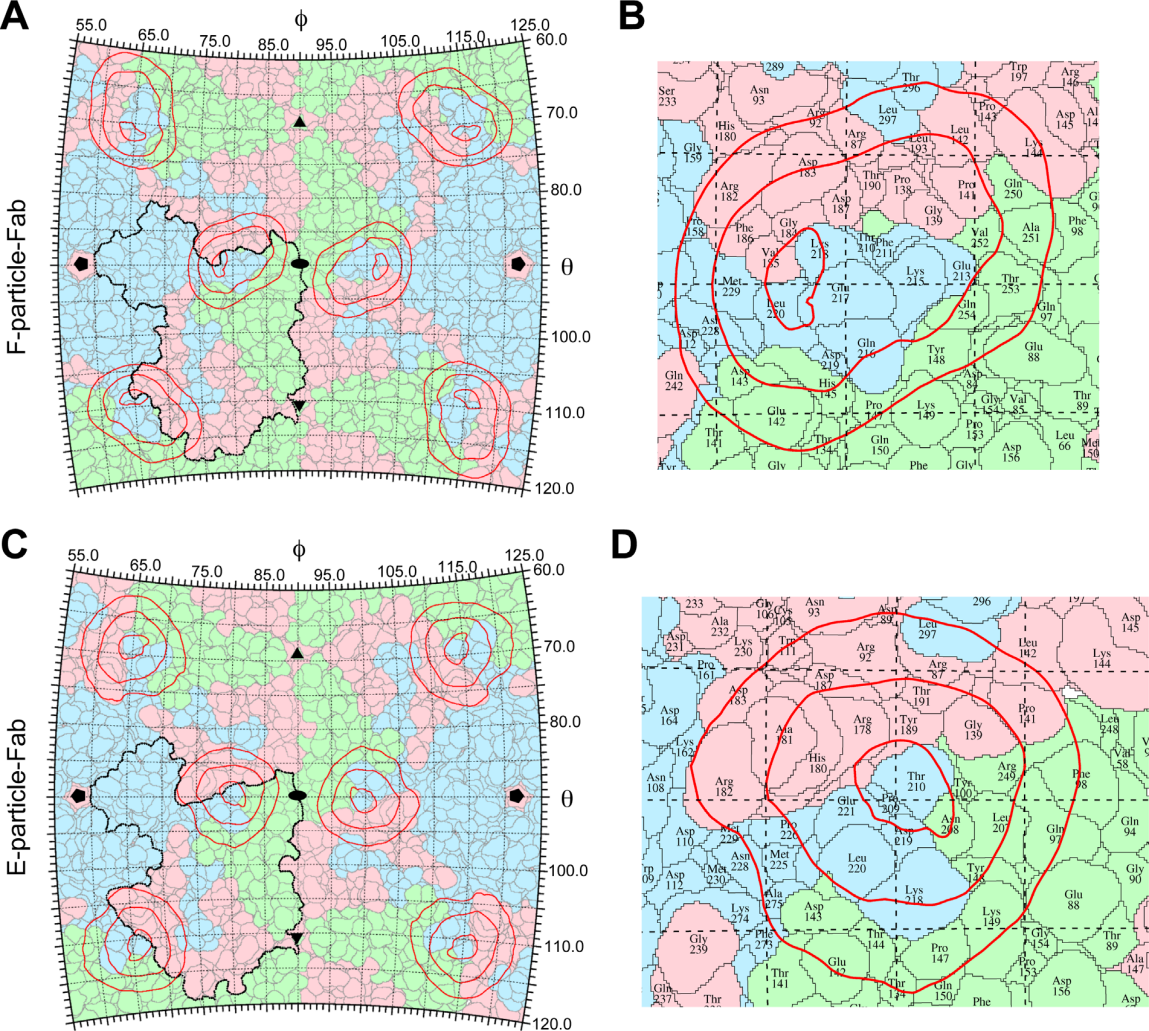
D5 Fab footprints on the surface of the EV71 particles. **(A-B)** Overall and expanded views of the D5 Fab footprints on the F-particle. The surface of the EV71 F-particle is shown as a stereographic projection, in which the polar angles θ and ɸ represent latitude and longitude, respectively. The D5 Fab footprints are indicated by red contour lines. The border of one VP2 (VP0)/VP3/VP1 protomer is outlined by black line. The locations of the 2-fold, 3-fold and 5-fold icosahedral symmetry axes are indicated as black ovals, triangles, and pentagons, respectively. In the expanded view, the amino acid residues of EV71 are denoted. The VP1, VP2 (VP0) and VP3 surfaces are shown in light blue, light green and pink, respectively. **(C-D)** Overall and expanded views of the D5 Fab footprints on the E-particle.

We determined the footprints of D5 on the EV71 virus by projecting the Fab density onto the surface of the F-particle (Fig 3A and 3B) as well as the E-particle (Fig 3C and 3D). The footprint of the Fab on the F-particle is slightly different from that on the E-particle, yet they both cover the interface between the two adjacent protomers at the same side of the 2-fold axis (Fig 3A and 3C). According to these footprints, the viral amino acid residues that, in both the F-particle and E-particle, were determined to be covered by the binding of D5 include T210, K218, D219, and L220 of VP1, Y100, E142, D143, P147 and Y148 of VP2, and R87, R92, G139, G140, P141, R182 and D187 of VP3 (Fig 3B and 3D). Note that VP1 residues 211–217 were missing in the E-particle crystal structure (PDB ID: 3VBU), and therefore not shown as part of the D5 footprints; however, K215, Q216 and E217 of VP1 were determined in the F-particle crystal structure and therefore were included in the D5 footprints on the F-particle (Fig 3). Interestingly, some of the D5 footprint amino acid residues are also involved in the interaction of EV71 with its receptors, indicating the pre-occupancy of D5 may hinder the receptor binding to EV71.

### Binding interface between EV71 and MAb D5

To further inspect the D5 binding interface with EV71, we also built a pseudo-atomic model of D5 Fab using SWISS-Model [32]. Sequence alignment of the variable regions of the heavy chain and light chain of D5 with the corresponding MAb template reveals that the CDR3 domain of D5 heavy chain shares the lowest homology with its template (S6 Fig), which is consistent with the functional role of the IgG CDR3 domain as the antigen-binding site.

**Fig 4:**
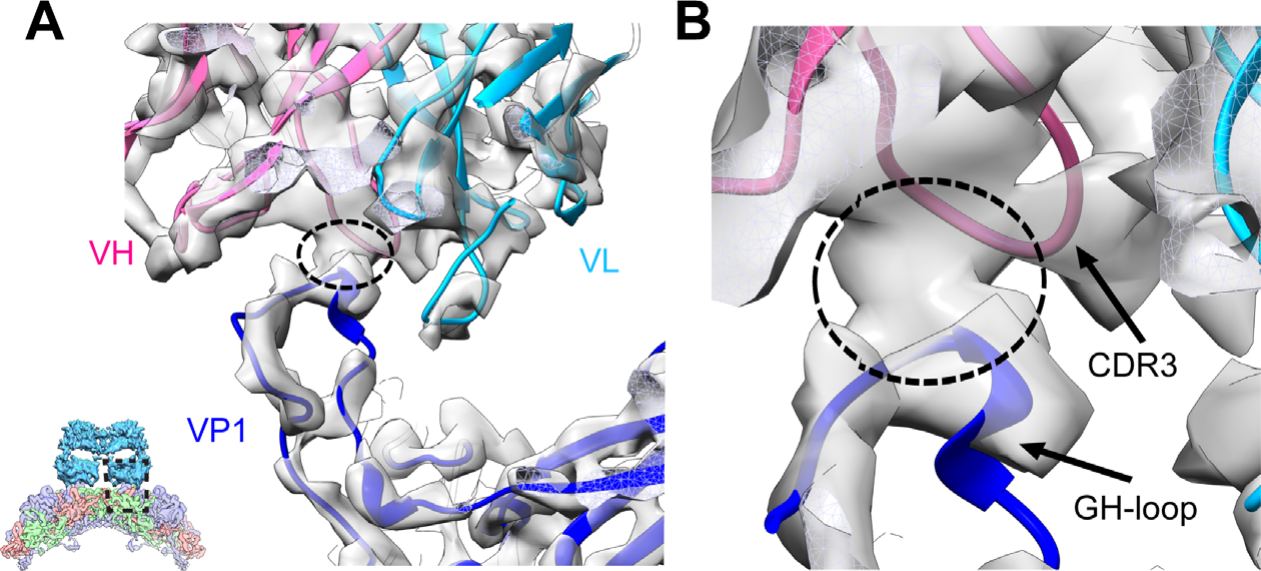
Binding interface between the D5 antibody and EV71 F-particle in the F-particle-Fab map. **(A)** Overall view of the D5-EV71 binding interface located between the D5 Fab variable region and the VP1 of EV71. Models of heavy and light chains of the Fab, and VP1 of EV71 are shown in light purple, cyan, and blue, respectively. Cryo-EM density is shown in grey. A solid density connecting the EV71 VP1 and the Fab heavy chain is highlighted by dotted black circle. The visualization location with respect to the F-particle-Fab map is illustrated using a small panel in the lower left corner. **(B)** An expanded view of the D5-EV71 binding interface. Black arrows point to the CDR3 region of D5 heavy chain and VP1 GH-loop of EV71, respectively.

In the 4.8 Å resolution F-particle-Fab map, the Fab densities in contact with capsid are well resolved, allowing us to fit the variable domain of the D5 Fab homology model into the map (Fig 4). The relative orientation of the variable regions of the heavy chain and light chain of D5 was determined based on the correlation score (0.642 versus 0.556), and the orientation with the higher score (Fig 4A) was chosen for further analysis. In this orientation, the variable region of heavy chain is facing the 2-fold axis, and all the CDR loops fit into the density unambiguously. Examination of the modeled Fab-virus interface reveals a solid piece of density connecting the EV71 VP1 GH-loop and the CDR3 region of D5 heavy chain (marked by the dotted black circle in Fig 4), suggesting an interaction between these two components. Also, minor clashes were observed in the models between these two regions after rigid body fitting, which were eliminated by further flexible fitting (see Methods). Taken together, our structural analyses combining the map and model information suggest that the interaction between D5 and EV71 involves the CDR3 region of D5 heavy chain and the VP1 GH-loop of EV71.

### Verification of the participation of heavy chain CDR3 in the binding of D5 to EV71

**Fig 5:**
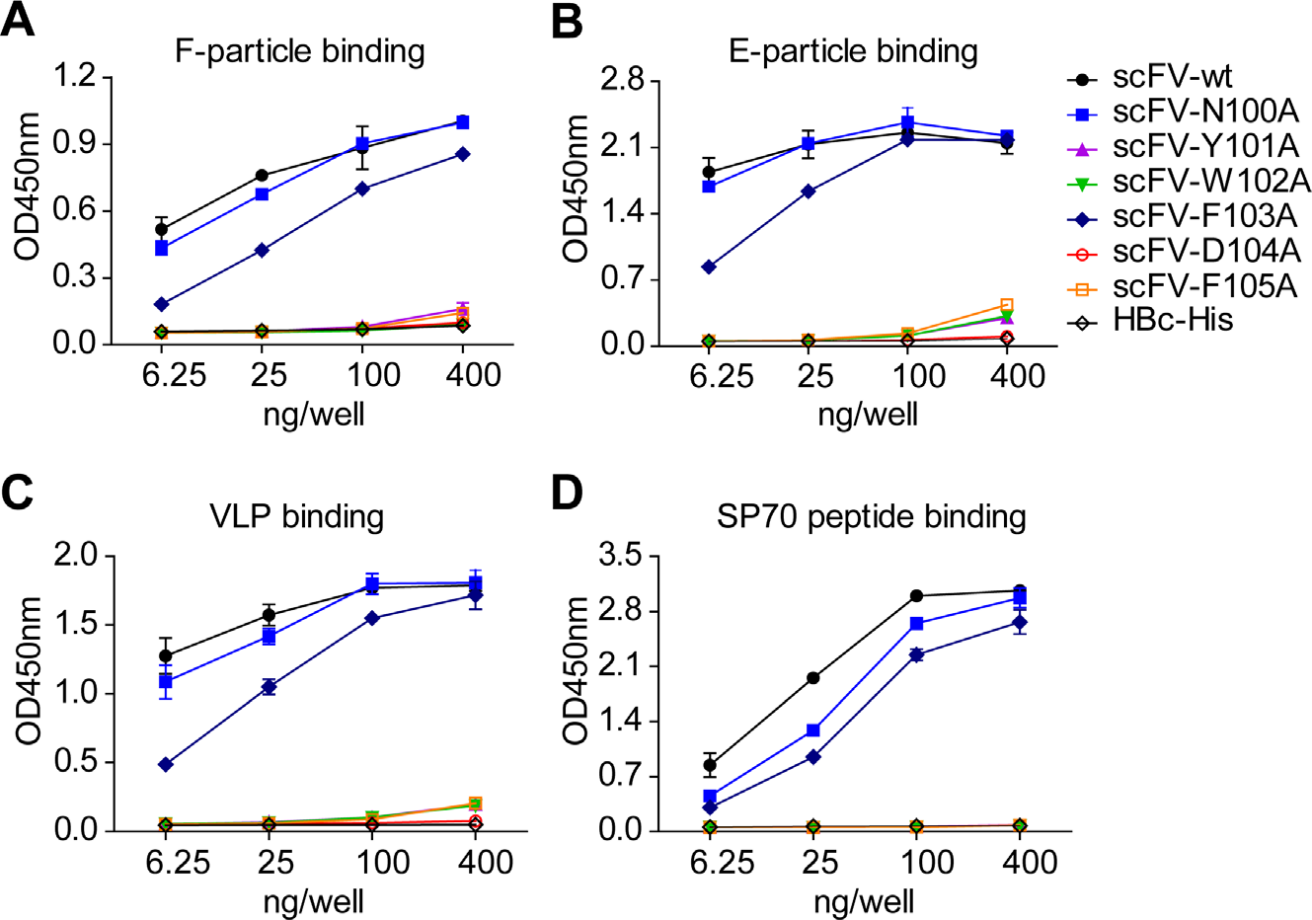
Effect of mutation of heavy chain CDR3 on D5 scFv binding to EV71 particles and SP70 peptide. The binding of D5 scFv variants with point mutations at heavy chain CDR3 region to **(A)** F-particle, **(B)** E-particle, **(C)** VLP, and **(D)** SP70 peptide were determined by ELISA assay. The irrelevant hepatitis B core protein (HBc) was used as negative control. Mean values and standard deviations of duplicate samples are shown.

To determine whether the CDR3 of heavy chain indeed contributes to the binding of D5 to EV71, we generated variants of a single-chain variable fragment (scFV) of D5 with point mutations in the heavy chain CDR3 loop, and evaluated their reactivities with different EV71 particles and the SP70 peptide (amino acid residues 208 to 222 of EV71 VP1) [33] corresponding to the GH-loop. As shown in Fig 5, wild-type scFv (scFv-WT) efficiently bound to the F- and E-particles, VLP, and the SP70 peptide. While the point mutant scFv-N100A did not significantly affect the binding capacity to all antigens tested, the mutant scFv-F103A displayed reduced binding capacity to different extents, and the other mutations, including Y101A, W102A, D104A and F105A, resulted in significantly diminished binding capacities. Altogether, our structural and biochemical results indicate that the heavy chain CDR3 loop of D5 is indeed involved in the recognition of EV71.

### Verification of the GH-loop of VP1 as the D5 binding epitope

**Fig 6:**
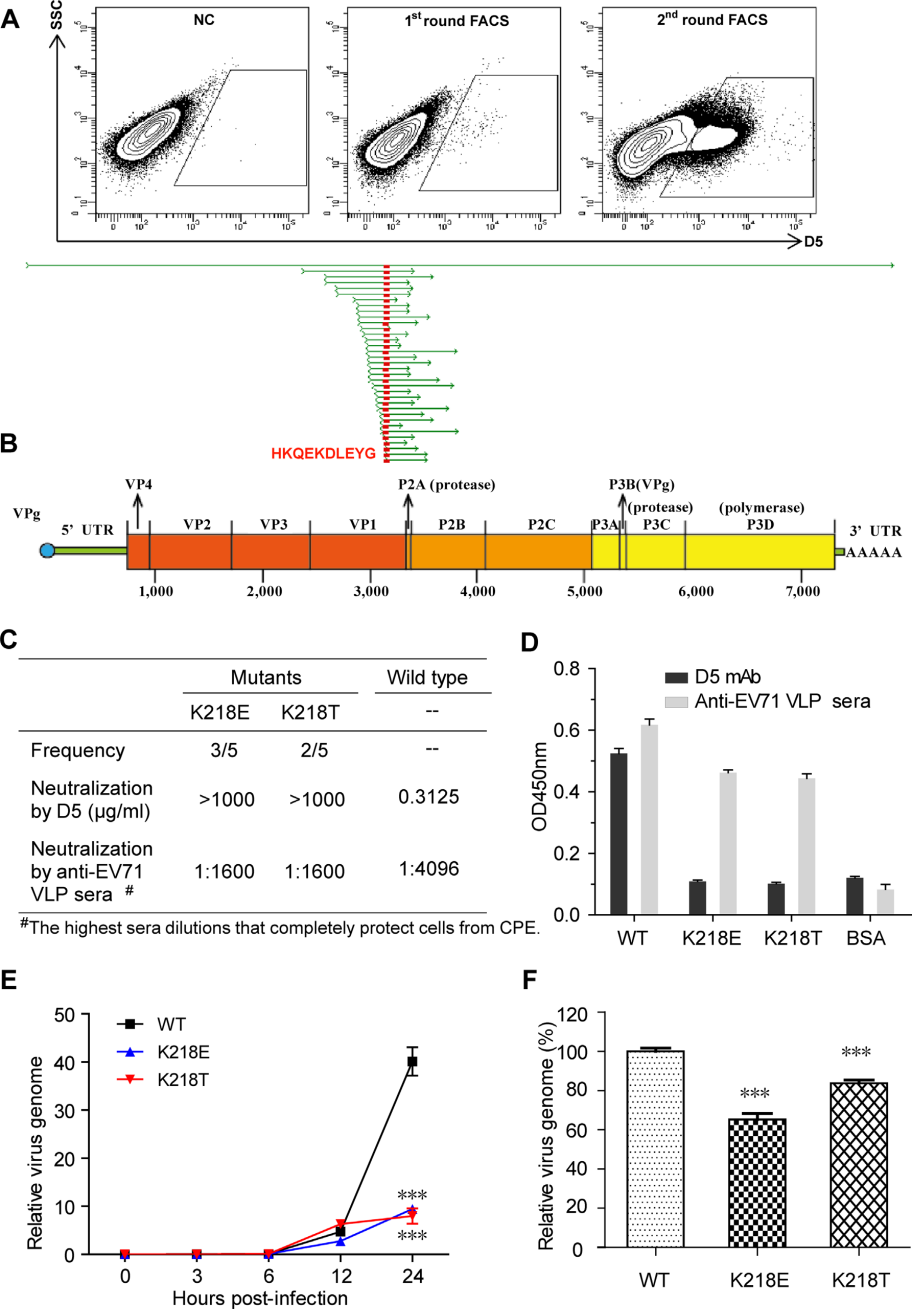
Mapping of the D5 epitope to VP1 region and identification of critical residue for D5 binding. **(A)** A yeast library expressing a combinatorial library of the entire polyprotein of EV71 on the surface was screened for D5 binding by fluorescence activating cell sorter (FACS) analysis. D5-positive yeast clones were isolated, and 34 of them were sequenced for the insertion. This panel shows the representative results of two rounds of sorting. **(B)** Alignment of the sequences obtained from the D5-positive yeast clones. The consensus region (HKQEKDLEYG) encoded by the VP1 gene is highlighted in red. **(C)** D5-resistant mutants were generated by passage of EV71/G082 in the presence of D5 antibody (1 mg/ml) and subsequent plaque purification. Five independent D5-resistant clones were recovered. This panel shows the susceptibility of wild-type and D5-resistant viruses to neutralization by D5 or anti-EV71 VLP mouse sera. **(D)** Binding activity of wild-type and D5-resistant viruses to D5 or anti-EV71 VLP mouse sera determined by ELISA. An irrelevant protein, BSA, serves as the negative control in the assay. Mean values and standard deviations of triplicate wells are shown. **(E)** Fitness of the D5-resistant mutants. Same amount (viral RNA genome copy number) of mutant or wild-type viruses was added to RD cells and incubated at 37°C for periods of time as indicated. The data are relative values of viral RNA copy normalized with GAPDH mRNA copy for each treatment. Means ± SD of triplicate wells were shown. Statistical significance was analyzed by two-way ANOVA using GraphPad Prism version 4. **(F)** Binding ability of D5-resistant mutants to SCARB2. Same amount of mutant or wild-type viruses was incubated with SCARB2-Fc and anti-human Fc IgG conjugated beads at 4°C as described in Methods. The pulled-down viruses were quantified by qRT-PCR. Y axis indicates the percentage of the viral RNA copy number of D5-resistant mutants to that of the wild-type virus. The data are Means ± SD of triplicate wells. Statistical significance was analyzed by the Student’s *t*-test using GraphPad Prism version 4.

Based on the fitted structure, the VP1 GH-loop of EV71 forms a direct contact with the heavy chain CDR3 of D5 (Fig 4). To further verify this structural finding, we carried out yeast display analysis [34] to map the D5 epitope. Yeast cells displaying a combinatorial library of the polypeptide fragments of EV71 were incubated with D5, followed by FACS analysis and cell sorting. Sequencing of the fragments isolated from positive yeast clones showed that the D5-reactive fragments derived in part or entirely from the VP1 of EV71 (Fig 6A and 6B). Alignment of the obtained sequences further revealed the consensus sequence HKQEKDLEYG (amino acid residues 214 to 223, Fig 6A and 6B), which is located within the GH-loop of VP1, consistent with the binding-interface analysis (Fig 4). These data provide additional evidence for the D5 binding epitope residing in the VP1 GH-loop of EV71.

We generated five independent EV71 mutants that can escape the neutralization by a high concentration (1 mg/ml) of D5. Genome sequencing in the P1 structural region of these mutants revealed that all five mutant viruses had a single amino acid change at the position of K218 within the VP1 GH-loop (Fig 6C). These escape mutants failed to bind D5, whereas they still reacted efficiently with a mouse anti-EV71-VLP polyclonal antibody (Fig 6D). Neutralization assays showed that these mutant viruses were resistant to 1 mg/ml of D5 but remained sensitive to the 1:1,600 diluted anti-EV71 VLP serum (Fig 6C). These results further verified that K218 within the D5 VP1 GH-loop is important for the susceptibility of EV71 to D5 binding and neutralization. Interestingly, we found that the growth of the mutants in RD cells is slower than that of the wild-type virus by 5 to 6 times (Fig 6E), indicating impaired fitness for the mutants. Moreover, in vitro pull-down assays showed that the mutants bound SCARB2 less efficiently than did the wild-type virus (Fig 6F), implicating that K218 is also involved in the interaction between EV71 and SCARB2.

### Broad neutralization capability of the D5 antibody

Notably, the VP1 GH-loop, here identified as the D5 binding epitope, also harbors a previously identified neutralizing epitope SP70 (amino acids 208–220) that is identical among all EV71 subgenotypes [33], suggesting that D5 has a broad neutralization potential. We thus evaluated the neutralization breadth of D5 using a panel of enteroviruses. This antibody was found to efficiently neutralize all of the tested clinical isolates of the EV71 subgenotype C4, the prototype BrCr strain belonging to genotype A, and a mouse-adapted EV71 strain termed EV71/MAV-W, with IC50s ranging from 0.183 to 1.592 μg/ml (Table 1). In contrast, no neutralization effect by D5 was observed on the other two HFMD-causing enteroviruses, CA16 and CA10, even at a concentration as high as 80 μg/ml. Taken together, these data demonstrate that D5 is a broadly neutralizing antibody against EV71.

**Table 1:** Neutralization activity of D5 on a panel of enteroviruses.

Virus strains	EV71 Subgenotypes	Neutralization concentration ^a^ (μg/ml)	IC50 ^b^ (μg/ml)
EV71/BrCr	A	0.625	0.233
EV71/G081	C4	0.625	0.219
EV71/G082	C4	0.313	0.183
EV71/FY573	C4	1.25	0.790
EV71/SZ98	C4	0.625	0.326
EV71/MAV-W	Mouse-adapted	2.5	1.592
CA16/SZ05	-	>80	ND
CA10/M.K.	-	>80	ND

^a^ Neutralization concentration was determined as the lowest antibody concentration required to fully prevent CPE that is observed by eye.

^b^ IC50 was determined as the lowest antibody concentration that inhibits 50% cell death as measured by an MTT assay.

ND, not determined.

### Protective efficacy of the D5 antibody

**Fig 7:**
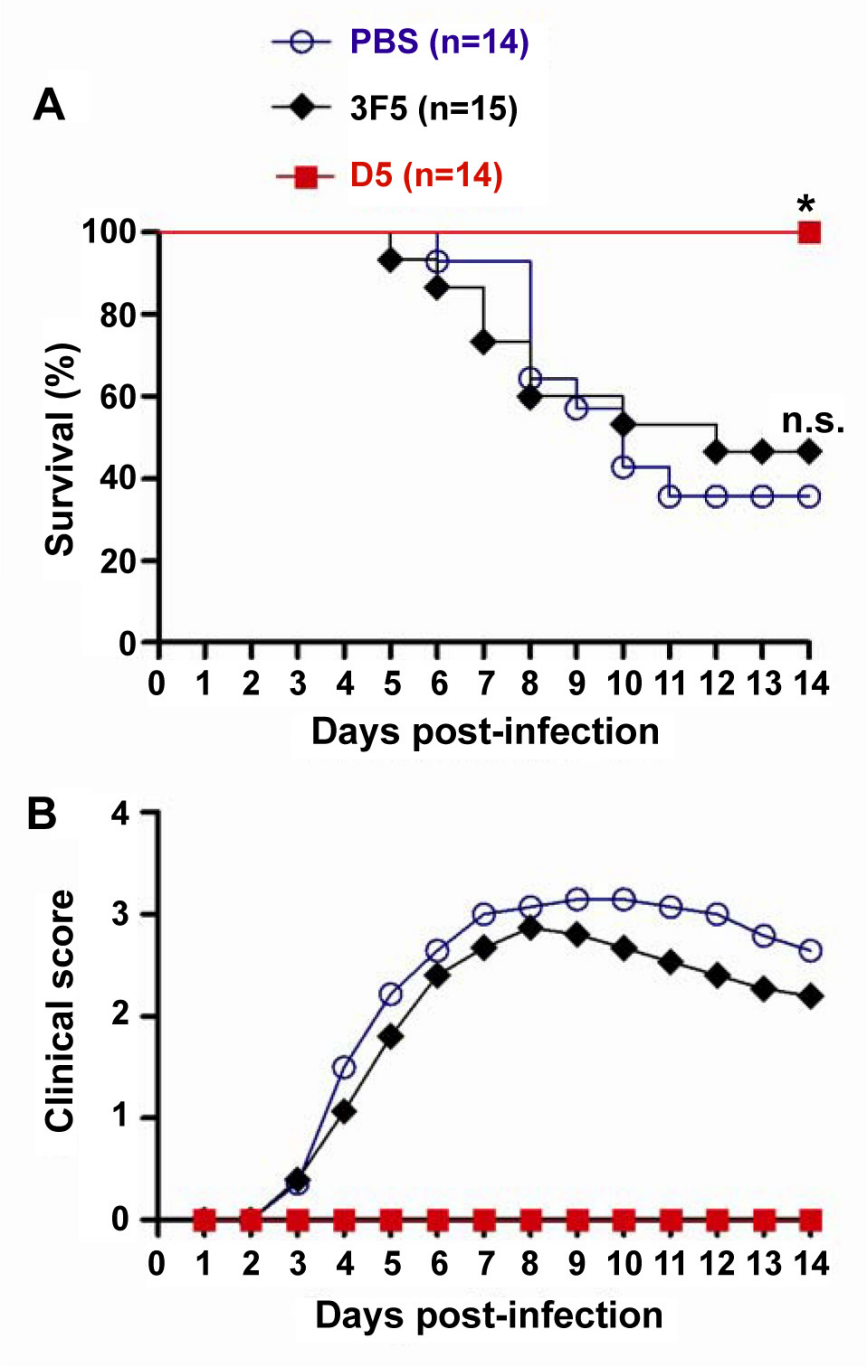
*In vivo* protective efficacy of antibody D5. Groups of mice were administered i.p. with PBS or the indicated mAbs, and one day later, inoculated i.p. with 5 × 10^5^ TCID_50_ of EV71/MAV-W. The mice were monitored daily for **(A**) survival and **(B)** clinical signs for a period of 14 days. Clinical scores were graded as follows: 0, healthy; 1, reduced mobility; 2, limb weakness; 3, paralysis; 4, death. The data shown are survival rates and mean clinical scores for each group at the indicated time points. The logrank test was used to compare the survival rate between each mAb group and the PBS control group. Statistical significance is indicated as follows: n.s., *P* ≥ 0.05; *, *P*<0.05; **, *P*<0.01.

The *in vivo* protective efficacy of D5 antibody was assessed in a mouse model of EV71 infection. This model is based on EV71/MAV-W, a mouse-adapted EV71 strain which is able to efficiently infect 7-day-old mice via the i.p. route [35]. Groups of 6-day-old mice were administered a single dose (10 μg/g body weight) of antibody or PBS, followed by inoculation i.p. one day later with EV71/MAV-W. Subsequently, the mice were monitored on a daily basis for survival and clinical signs. As shown in Fig 7, the control mice administered PBS or an irrelevant monoclonal antibody 3F5 gradually developed clinical signs, including reduced mobility, limb weakness and paralysis, and eventually died with a final mortality rate of 64.3% and 53.3%, respectively, by day 14 after infection. In contrast, 100% of the mice that had received mAb D5 survived without notable clinical signs during the whole 14-day period. These results indicate that D5 treatment fully protected mice from EV71 infection.

## Discussion

To thoroughly understand the structural basis for recognition of EV71 by a broadly neutralizing antibody D5, we determined four cryo-EM structures on the immune complexes of EV71 with the D5 intact IgG or Fab all at subnanometer resolution. We found that the intact D5 IgG binds to EV71 virion in a bivalent manner. Specifically, our high-resolution cryo-EM maps reveal that D5 IgG bivalently binds the surface exposed VP1 GH-loops over the 2-fold axis on EV71 virions. Previously, a human rhinovirus neutralizing antibody (named mAb17-IA) targeting the VP1 BC-loop was reported to bind the virus bivalently across the icosahedral 2-fold axis [36]. In addition, an anti-poliovirus monoclonal antibody C3 have been suggested to bivalently bind two adjacent VP1 BC-loops on the same pentameric “mesa” but not across the 2-fold axis, based on a 11 Å cryo-EM structure of the poliovirus-C3 Fab complex [37]. Clearly, as compared to the mAbs 17-IA or C3, D5 adopts a unique bivalent binding mode in terms of its epitope and binding pattern. The present and previous studies thus reveal the diversity of bivalent binding modes of neutralizing antibodies to picornaviruses.

The bivalent binding of intact D5 IgG on the EV71 F-particle (Fig 2) could result in a cross-linking of two adjacent protomers over the 2-fold axis, which may therefore stabilize the virion. It has been suggested that the hole at the 2-fold axis in the A-particles (the uncoating intermediate form of EV71) increases in size upon being heated and serves as a channel through which the viral RNA genome is released [31]. Therefore, it is possible that the cross-linking by D5 IgG over the 2-fold axis may restrict the size expansion of the channel and block the release of viral RNA, thereby providing an additional neutralization effect as compared to that provided by the binding of the monovalent Fab. Indeed, we found that the intact IgG of D5 was much more potent in neutralizing EV71 than its Fab fragment (S5 Fig), i.e., the bivalent intact IgG of D5 was found to be ~19 times more potent than was the monovalent Fab in the post-attachment neutralization assay whereas the avidity of IgG to the F-particle was only 2.5 times higher than that of Fab. These data strongly suggest that the bivalent binding of D5 IgG may neutralize EV71 by restricting conformational changes of the capsid required for release of viral RNA.

Enterovirus entry involves virus attachment to target cells, internalization and the receptor mediated uncoating and release of viral RNA. The molecule SCARB2 has been identified as an uncoating receptor for EV71 [9]. Although the exact binding site for SCARB2 on EV71 has not been accurately identified, GH-loops of VP1 and VP3 of EV71 have been shown to interact with SCARB2 [38]. Interestingly, in the present study, we identified the VP1 GH-loop as the binding epitope of D5; meanwhile, the footprint of D5 on the F-particle also covers the GH-loop of VP3 (R182, D183, and G184) (Fig 3B). Therefore, upon binding EV71, D5 may preoccupy the SCARB2-binding site and thus inhibit uncoating of EV71. In addition, the footprint of D5 also covers K149 of VP2 (Fig 3B), which has been shown to be critical in the binding of EV71 to another identified receptor, PSGL1 [10,39]. Thus, it is conceivable that the binding of D5 to EV71 may sterically hinder access of PSGL1 to its binding site. In agreement with the above speculations, it has been recently reported that pretreatment of EV71 with D5 was indeed able to inhibit the binding of SCARB2 as well as of PSGL1 to EV71 in an antibody dose-dependent manner [40]. Moreover, in the present study we found that D5-resistant mutant viruses carrying a single mutation at the position of K218 within the VP1 GH-loop exhibited impaired binding to SCARB2 (Fig 6F), indicating that the K218 residue, as a part of the D5 epitope, is involved in the interaction between EV71 and SCARB2. Overall, our data reveal that inhibition of receptor binding on EV71 is likely one of the neutralization mechanism of D5.

In summary, using cutting-edge cryo-EM technology in combination with a number of biochemical and virological approaches, we have elucidated the structural basis for recognition of EV71 by the highly potent antibody D5. Our studies clearly depict a unique bivalent binding pattern of intact D5 IgG on the VP1 GH-loop of the EV71 F-particle. Based on our results, we propose that D5 neutralizes EV71 infection through two mechanisms, including the blocking of receptor binding and the potential inhibition of conformational changes of the EV71 capsid. In addition, our results demonstrate that D5 is broadly neutralizing *in vitro* and highly protective *in vivo*. These findings should enhance our understanding of antibody-based protection against EV71 infections and facilitate the development of D5-derived anti-EV71 drugs.

## Materials and Methods

### Cells and viruses

RD (ATCC, CCL-136) and Vero (ATCC, CRL-1586) Cells were maintained as described previously [14]. EV71 strains used in this study include EV71/BrCr (ATCC, VR-1775), EV71/G081, EV71/G082, EV71/FY2, EV71/SH98, and a mouse-adapted virus termed EV71/MAV-W, which have been described previously [35]. CA16/SZ05 (GenBank accession no. EU262658) and CA10/M.K. (Kowalik strain, ATCC, VR-168) strains have been described previously [15]. All viruses were titrated for the 50% tissue culture infectious dose (TCID50) as described previously [41].

### Preparation of inactivated EV71 particles

Inactivated EV71 particles were prepared as described previously [42]. The resulting inactivated EV71 preparations were assessed for purity by SDS-PAGE and Western blotting as described previously [43]. Cryo-EM microscopy revealed that the inactivated EV71 preparations contain both immature empty particles (E-particles) and mature virions (F-particles) (S1A and S1B Fig). In addition, E- and F-particles were prepared separately as described previously [14].

### Preparation of EV71 VLP

Recombinant VLP were produced in transgenic *Pichia pastoris* yeast as described previously [35]. The final VLP preparation was analyzed by SDS-PAGE, Western blotting and ELISA as described previously [15].

### Preparation of D5 IgG, Fab and scFv

D5 IgG was purified as previously described [20]. To prepare the Fab fragment, D5 IgG antibody was digested with papain at a weight ratio of 200:1 in 100 mM phosphate buffer (pH 7.0) containing 20 mM L-Cys and 1 mM EDTA for 10 h at 37°C before adding 25 mM iodoacetamide to stop the reaction. The resulting Fab fragment was purified using a Hitrap Protein G column (GE Healthcare, NJ, USA) followed by size-exclusion chromatography with a Sephacryl S-100 column (GE Healthcare). Purified D5 Fab monomer was quantified by using a Bradford assay and stored at -80°C before use.

To clone the genes encoding the D5 antibody, RNA was isolated from D5 hybridoma cells using Trizol (Invitrogen) and converted to cDNA by reverse transcription. The variable regions of the heavy chain and light chain were amplified by PCR using 5’ RACE system (Invitrogen) according to the manufacturer’s instructions. The final PCR products were cloned into the pGEM-T vector (Promega) for sequence determination. To produce a wild-type single-chain antibody fragment (scFv), coding sequences for variable regions of heavy chain (V_H_, 1–118 aa) and light chain (V_L_, 1–113 aa) were joined through an 11-amino acid residue (GGGGS)_2_G linker by overlap PCR. The resulting PCR product, designated V_H_-(GGGGS)_2_G-V_L_, was ligated into pET28b to yield the plasmid pET28b-D5-scFv-wt. To produce the scFV mutants, point mutations (N100A, Y101A, W102A, F103A, D104A or F105A of V_H_) were engineered into the plasmid pET28b-D5-scFv-wt by overlap PCR. The resulting plasmids were verified by sequencing. For scFv expression, the plasmids were individually transformed into *E*. *coli* strain BL21. After induction, the transformed *E*. *coli* cells were lysed by sonication. The His-tagged target protein was purified from the soluble fractions of the lysate using a Ni-NTA resin. Purified scFv was quantified by the Bradford assay.

### Cryo-EM imaging

Immune complexes were prepared by mixing EV71 VLP (1.4 mg/ml) or inactivated virus (0.8 mg/ml) with 1.2-times saturated D5 antibody or Fab fragment, equivalent to a molar ratio of 1:72, and then incubated at 37°C for 2 h. An aliquot of a 2 μl sample was deposited onto a glow discharged holey carbon Quantifoil Cu grid (R1.2x1.3, 200 mesh, Quantifoil Micro Tools), which was covered with a thin layer of home-made continuous carbon film. After 2 s blotting to remove extra sample, the grid was plunge-frozen into liquid ethane using a FEI Mark IV Vitrobot. Specimens were examined under low-dose conditions at 300 kV with a FEI Titan Krios transmission electron microscope equipped with a Cs corrector. Images were recorded on a Falcon II direct electron detector in the 7-frame movie mode. The electron dose rate was set to ~16 e^−^/Å^2^・s and the exposure time was 1.1 s. All the images were recorded at a nominal magnification of 37,000, corresponding to a pixel size of 1.79 Å, and with the defocus ranging from 2 to 3.5 μm.

### Cryo-EM single particle 3D reconstruction

To correct the drift and beam-induced motion, the 7 frames in each movie were aligned to generate a single micrograph using Motioncorr [44]. Due to the sticky nature of the sample, particles were manually boxed using the *e2boxer*.*py* program from the EMAN2.1 package [45]. CTF fitting was automatically performed using the *fitctf2*.*py* program in jspr package [46], then visually validated and adjusted using EMAN1.9 *ctfit* program [47]. The structure factor was determined by computational fitting of multiple micrographs (http://blake.bcm.edu/emanwiki/EMAN1/FAQ/StructureFactor). Reference-free 2D analysis and initial model building were performed in EMAN2.1 [45]. The gold standard 3D reconstruction procedure was followed using the jspr package [21], with the datasets split into two halves in the very beginning of the iterative refinement. The jspr package was developed on the basis of EMAN1 and EMAN2, but more customized to handle virus system with great gain in efficiency. The further refinement of defocus, astigmatism and magnification of individual particle was all carried out in jspr [21]. The map resolutions were assessed in jspr [21] using gold standard criteria of 0.143 FSC cutoff. After reconstruction, the maps were sharpened with structure factors to boost the density map Fourier amplitudes, and then low pass filtered to the assessed overall resolution using *e2proc3d*.*py* in EMAN2.1 [45]. To better visualize and compare the Fab/IgG densities, usually at relative lower resolution than that of the capsid region, we also showed the original unsharpened maps that is usually more dominated by low-resolution features [48] (S4 Fig). The absolute pixel size and handedness were confirmed by the good fitting of EV71 mature virus and empty particle crystal structures (PDB ID: 3VBS and 3VBU) into the corresponding maps using UCSF Chimera [49].

Local resolution was estimated using ResMap [22]. The local resolution evaluation of the F-particle-Fab map reveals that the resolution of the Fab/IgG portion, especially that of their distal region, is lower than that of the capsid portion (S3 Fig), which is consistent with a previous cryo-EM study on virus-Fab complexes [26]. This can be mainly attributed to the intrinsic dynamic nature of Fab/IgG especially of their distal region. In addition, the potential sub-stoichiometric occupancy of Fab/IgG on the particle surface could also contribute to the overall lower resolution of the Fab/IgG compared to that of the capsid.

### D5 Fab homology model building

There is no atomic-resolution structure available for antibody D5 yet. We thus carried out a sequence Blast search for the variable regions of the heavy and light chains of antibody D5. Two structures, the heavy chain of the R218 antibody (PDB ID: 4K2U) [50] and the light chain of the MAb 1479 (PDB ID: 3U9U) [51], which share high sequence identity with their counterparts in D5 (S6 Fig), were chosen as templates. The heavy chain of D5 shares 85% sequence identity with that of R218, with similar CDR1 and CDR2 regions but divergent CDR3 regions (S6A Fig). The light chain of D5 shares 93% sequence identity with that of MAb 1479, and they have identical CDR1 and CDR2 regions and also similar CDR3 regions (S6B Fig). The subsequent homology model building was performed through the SWISS-Model server [32].

### Fitting of Fab and Roadmap calculation of Fab Footprint

Crystal structures of EV71 mature and immature virus (PDB code: 3VBS and 3VBU) [5] were fitted into the corresponding maps utilizing UCSF Chimera’s *Fit in Map* module [49]. Since the distal end of the Fab density was less well resolved due to its high flexibility, only the variable domain of this D5 Fab model was fitted into the corresponding map as a rigid body, also using *Fit in Map* module in Chimera [49]. This rigid-body fitting resulted in minor clashes between the CDR3 loop of D5 heavy chain and the GH-loop of EV71 VP1. We then carried out further flexible fitting on the models of the variable domains of the heavy and light chains of D5 Fab together with the contacting VP1 of EV71 with the restraint of the cryo-EM density map, using the molecular dynamics flexible fitting (MDFF) program [52]. Only the heavy chain variable domain of D5 Fab was allowed to move while the other portions remained fixed in this procedure.

The Fab density in the difference maps were projected on a stereographic sphere using RIVEM [53]. The D5 Fab footprints were generated by projecting the radial density corresponding to the interface between D5 Fab and the EV71 particles onto the particle surface. The atomic coordinates from EV71 F-particle or E-particle crystal structures are represented as roadmap.

### Binding ELISA

For binding ELISA, 96-well plates were coated with 20 ng/well of EV71 E-particle, F-particle or VLP, or 100 ng/well of SP70 peptide diluted in PBS buffer, and subsequently incubated at 4°C for 12 h. The plate was washed five times with PBST buffer (PBS with 0.05% Tween-20) after the above and each of the following steps. The wells were blocked with 200 μl/well of 5% nonfat milk in PBST for 1 h at 37°C, incubated with 50 μl/well of serially diluted scFv antibody or the irrelevant protein HBc for 2 h at 37°C, and then incubated with 50 μl/well of HRP-conjugated anti-His monoclonal antibody (ProteinTech) for 1 h at 37°C. After color development, the absorbance was determined at 450 nm in a 96-well plate reader.

### *In vitro* neutralization assay

The standard neutralization assay was performed as described previously [20]. Briefly, 100 TCID50 of EV71 G082 virus was incubated with serially diluted D5 IgG or Fab in a 96-well plate at 37°C for 1 h, then 1.5*10^4^ of RD cells in 100 μl medium were added to each well and cultured at 37°C for 72 h. The cell viability was measured by using a MTT assay. The percent of protection was calculated using the following formula: protection% = (OD490 of a given sample－average OD490 of virus only control) / (average OD490 of cells only control－average OD490 of virus only control)*100. The 50% inhibition concentration (IC50) of D5 IgG and Fab were calculated using GraphPad Prism 5.0.

Post-attachment neutralization assay was performed as described previously [43]. Briefly, 100 TCID50 of EV71 G082 virus was incubated with pre-seeded RD cells in 96-well plate at 4°C for 1 h followed by the washing away of the unbound virus. Next, serially diluted D5 IgG or Fab was added to the virus-bound cells and incubated at 37°C for 1 h; then the unbound D5 IgG or Fab was also washed away and the cells were cultured at 37°C for 72 h. The IC50 of D5 IgG and Fab were determined as described above.

### Bio-layer interferometry binding assay

The binding avidities of D5 IgG and Fab to the EV71 F-particle were analyzed by bio-layer interferometry on an Octet RED 96 system (ForteBio) in kinetics buffer (PBS buffer supplemented with 0.1% BSA and 0.02% Tween-20) at room temperature. The inactivated EV71 F-particles were labeled with biotin using the EZ-Link Sulfo-NHS-LC-LC-Biotin kit (Thermo Scientific). After a brief rinse in kinetics buffer, the streptavidin (SA) biosensor tips were dipped into 0.066 μg/ml of EV71-biotin solution for 10 min. Following a rinse in kinetics buffer, the EV71-immobilized SA biosensors were allowed to associate with D5 IgG or Fab at different concentrations (40, 8, 1.6, 0.32, 0.064 and 0.0128 μg/ml) for 25 min and then dissociate in kinetics buffer for 10 min. EV71-bound biosensor was also allowed to associate with kinetics buffer alone (without D5 IgG or Fab) to serve as a loading control. In addition, an empty sensor tip (without EV71) was allowed to associate with 40 μg/ml of D5 IgG or Fab to assess non-specific binding. Data were processed using Octet Data Analysis v6.4 (ForteBio).

### Yeast display assay

A yeast display technique described previously [34] was employed to map the D5 epitope. Briefly, a pool of yeast (*S*. *cerevisiae*) expressing a combinatorial library of the P1 protein of EV71 on the surface was constructed. After growth and induction, the yeast cells were mixed with D5 to allow binding. Afterwards, antibody-treated yeast cells were subjected to immunofluorescence staining and sorting of positive yeast by FACS. Finally, the plasmids from positive yeast clones were isolated and sequenced. The results were analyzed using Sequencher 4.9 (Gene Codes Corp., Ann Arbor, MI).

### Generation and sequencing of D5-escaping mutants

Briefly, antibodies (400 μg/ml) were mixed with 1×10^8^ TCID_50_ of EV71/G082 virus, and the mixtures were incubated at room temperature for 1 h and then at 37°C for 2 h. The virus/antibody mixtures were added to 2x10^6^ RD cells, followed by incubation for two days at 37°C. The cultures were harvested and subjected to three freeze-thaw cycles. Virus titer was determined by applying a micro-titration assay. After that, a second round of infection in the presence of D5 was performed as above except that the antibody concentration was increased to 1 mg/ml. After two rounds of selection, the resulting D5-resistant viruses were plaque purified on Vero cells. The P1 region of the plaque-purified, D5-resistant mutants was amplified by RT-PCR as described previously [43] using primers (forward 5’-GGCCATCCGGTGTGCAACAG-3’ and reverse 5’- AGCAAGTCGCGAGAGCTGTC-3’) and subsequently verified by sequencing.

### Fitness determination of the D5-resistant mutants

EV71 wild-type and mutant (K218E and K218T) viruses were quantified by qRT-PCR assay. A plasmid pIEXBac-P1 which contains the entire P1 gene of EV71 [43] was used as the standard for calculation of viral genome copies. To determine the fitness, same amount of viruses (containing 9.45 x 10^6^ copies of viral RNA genome) was added to 3 x 10^5^ RD cells preseeded 1 day ahead in a 24-well plate and incubated at 4°C for 1 h. After removing unbound virus, fresh medium were added to the cells and cultured at 37°C. Samples (both medium and cells) were collected at 0, 3, 6, 12 and 24 h post infection, respectively, and then subjected to RNA extraction. The resulting RNA was analyzed for viral genome copy number by qRT-PCR. GADPH mRNA of the samples was also determined, serving as the internal control. Data analysis was performed using the 2^-∆CT^ method.

### *In vitro* pull-down assay

The binding of EV71 variants to SCARB2 was determined by an in vitro pull-down assay. Briefly, same amount of the EV71 wild-type or mutant viruses (2.23 x 10^7^ copies of viral RNA genome per treatment) were mixed with 1 μg of SCARB2-Fc (Catalog 1966-LM-050, R&D) and 15 μl of anti-human Fc IgG conjugated agarose beads (Catalog A3316-5ML, Sigma) in 500 μl DMEM medium plus 2% FBS, and then incubated at 4°C for 3 h with gentle rotation. The unbound virus was removed by washing with PBS buffer. The virus-bound beads were treated with 200 μl of Trizol reagent and mixed thoroughly with 100 μl CHO lysate for total RNA extraction. The samples were analyzed for viral RNA genome and mouse β-actin mRNA (as the internal control) by qRT-PCR. Data analysis was performed using the 2^-∆∆CT^ method. The results were shown as the percentage of the EV71 genome copy numbers of the mutant viruses in relation to that of the wild-type one.

### *In vivo* protection assay

Groups of six-day-old suckling ICR mice were i.p. administered with PBS or a single dose (10 μg/g body weight) of the control mAb or D5 mAb. One day later, the mice were inoculated i.p. with 5×10^5^ TCID_50_ of EV71/MAV-W and then monitored daily for survival and clinical signs for a period of 14 days. Clinical scores were graded as follows: 0, healthy; 1, reduced mobility; 2, limb weakness; 3, paralysis; 4, death. The mouse experiments were approved by IACUC at Institut Pasteur of Shanghai. Animals were cared for according to the institutional guidelines.

### Accession numbers

The cryo-EM density maps of EV71 F-particle-Fab, F-particle-IgG, E-particle-Fab, and VLP-IgG have been deposited in the Electron Microscopy Data Bank under accession codes of EMD-6366, EMD-6365, EMD-6383 and EMD-6384, respectively. The model covering the EV71 VP1 GH-loop and the variable region of D5 Fab refined against the cryo-EM density map of F-particle-Fab was deposited in the Protein Data Bank under accession code of 3JAU.

## Supporting Information

S1 Fig Representative cryo-EM images. **(A)** EV71 (including both the E- and F-particles) in complex with D5 Fab. **(B)** EV71 (including both the E- and F-particles) in complex with intact D5 IgG. **(C)** VLP in complex with D5 IgG. The red arrows indicate the EV71-bound Fab or intact IgG. The black and white arrow-heads indicate F-particles and E-particles, respectively. Scale bar = 50 nm. These images also indicate the sticky nature of the samples.
(TIF)

S2 Fig Cryo-EM map resolution evaluations. **(A)** Resolution evaluation of the cryo-EM reconstructions by Fourier shell correlation (FSC) at 0.143 criterion. **(B)** The structural features of segmented VP1 compact regions (fitted model in blue) in F-particle-IgG, E-particle-Fab and VLP-IgG maps are shown.
(TIF)

S3 Fig Local resolution evaluation of the F-particle-Fab map. Local resolutions estimated by Resmap were rendered by four representative discrete 2D slides of the map. The color bar on the left labels the corresponding resolution (unit is Å), with the dark blue representing 4.5 Å and deep red representing 7.0 Å resolution.
(TIF)

S4 Fig Unsharpened cryo-EM density maps and corresponding cut-away views of the immune complexes. **(A)** F-particle-Fab complex. The complete particle is shown on the left. To better render the relative location of the bound Fabs/IgGs, the corresponding cut-away view of the central slice is also displayed on the right. **(B)** F-particle-IgG complex. (**C)** E-particle-Fab complex. **(D)** VLP-IgG complex. The same radial colour scheme from the centre of a sphere is used as in Fig 1A–1D. The icosahedral 5-fold, 3-fold and 2-fold symmetry axes are indicated in the cut-away view. The black arrow-heads on the left panels indicate a pair of adjacent Fab densities across the 2-fold axis, and the red arrows on the right panels indicate a hollow middle region between the two lobes in a Fab density.
(TIF)

S5 Fig Comparison of the neutralizing capacity and binding avidities of intact IgG and the Fab fragment of D5. **(A)** IC50s determined by the standard neutralization assay. **(B)** IC50s determined by the post-attachment neutralization assay. The error bars indicate standard deviations of triplicate wells at each concentration. IC50s were calculated by GraphPad Prism 5.0. **(C)** Bio-layer interferometry analysis of D5 IgG. (**D)** Bio-layer interferometry analysis of D5 Fab.
(TIF)

S6 Fig Sequence alignment of D5 Fab with corresponding templates. **(A)** In the variable region of heavy chain, antibody D5 shows high sequence identity with antibody K218 (PDB ID: 4K2U) in the CDR1 and CDR2 regions, but not in the CDR3 region. **(B)** In the variable region of light chain, D5 shows high sequence identity with antibody 1479 (PDB ID: 3U9U) in not only the CDR1 and CDR2 regions, but also the CDR3 region.
(TIF)

S1 Table Number of images and particles, and final resolution for the cryo-EM reconstructions. (DOCX)

S2 Table Correlation scores between cryo-EM density maps* of different immune complexes, calculated by the Chimera *Fit In Map* module. * For the correlation calculation, the entire density maps were used (including the IgG/Fab).
(DOCX)
