# Supplementary figures and images for: Structural Basis for Recognition of Human Enterovirus 71 by a Bivalent Broadly Neutralizing Monoclonal Antibody
Source: PLoS Pathog. 2016 Mar 3;12(3):e1005454. doi: 10.1371/journal.ppat.1005454 (PMC4777393; doi:10.1371/journal.ppat.1005454)

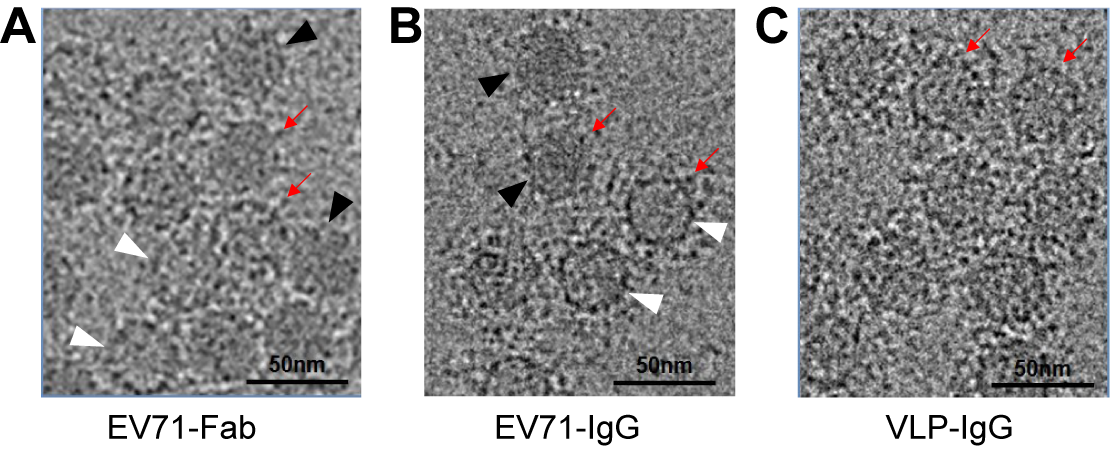

Supplement: S1 Fig — (A) EV71 (including both the E- and F-particles) in complex with D5 Fab. (B) EV71 (including both the E- and F-particles) in complex with intact D5 IgG. (C) VLP in complex with D5 IgG. The red arrows indicate the EV71-bound Fab or intact IgG. The black and white arrow-heads indicate F-particles and E-particles, respectively. Scale bar = 50 nm. These images also indicate the sticky nature of the samples. (TIF) [file ppat.1005454.s001.tif]

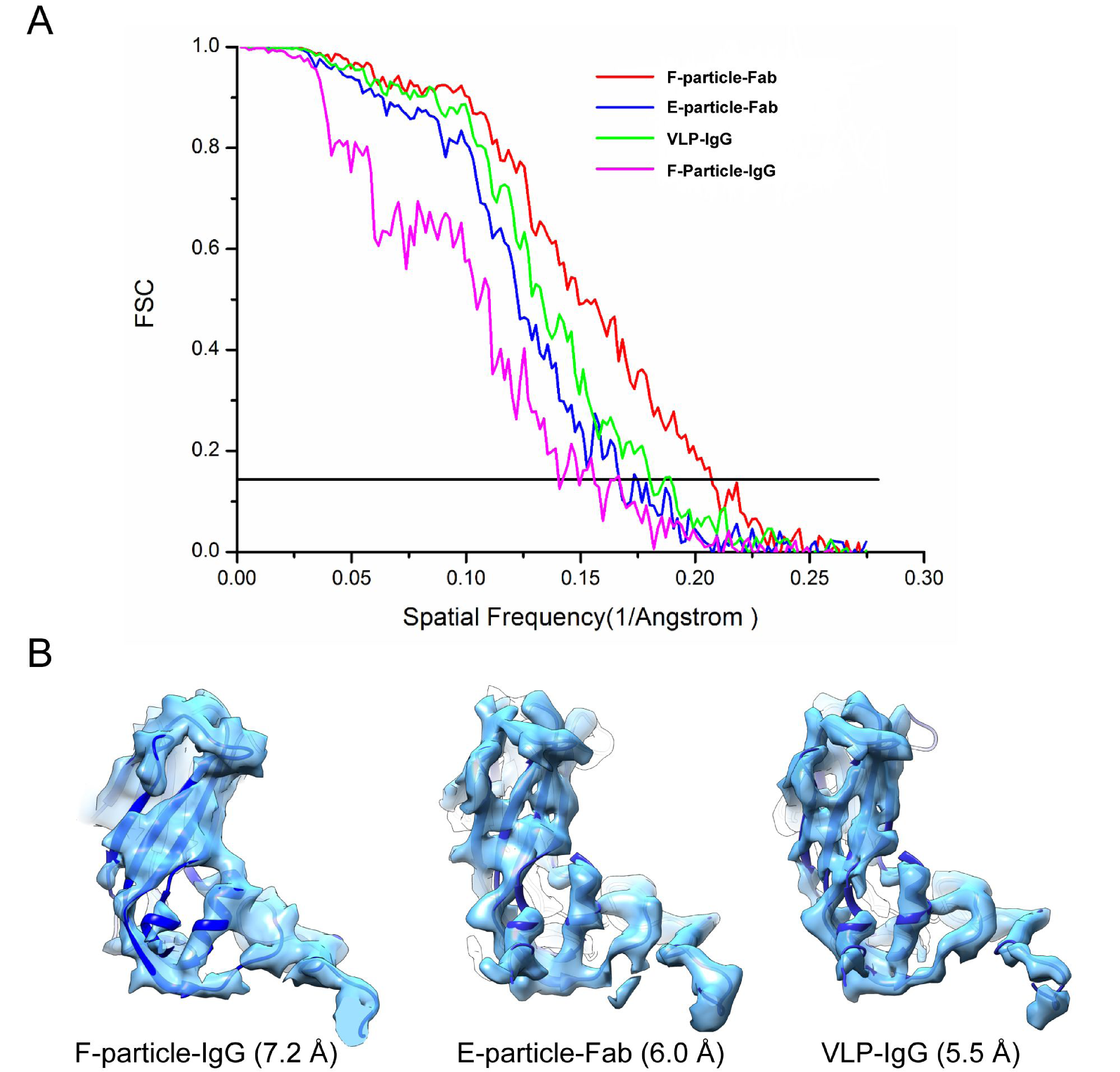

Supplement: S2 Fig — (A) Resolution evaluation of the cryo-EM reconstructions by Fourier shell correlation (FSC) at 0.143 criterion. (B) The structural features of segmented VP1 compact regions (fitted model in blue) in F-particle-IgG, E-particle-Fab and VLP-IgG maps are shown. (TIF) [file ppat.1005454.s002.tif]

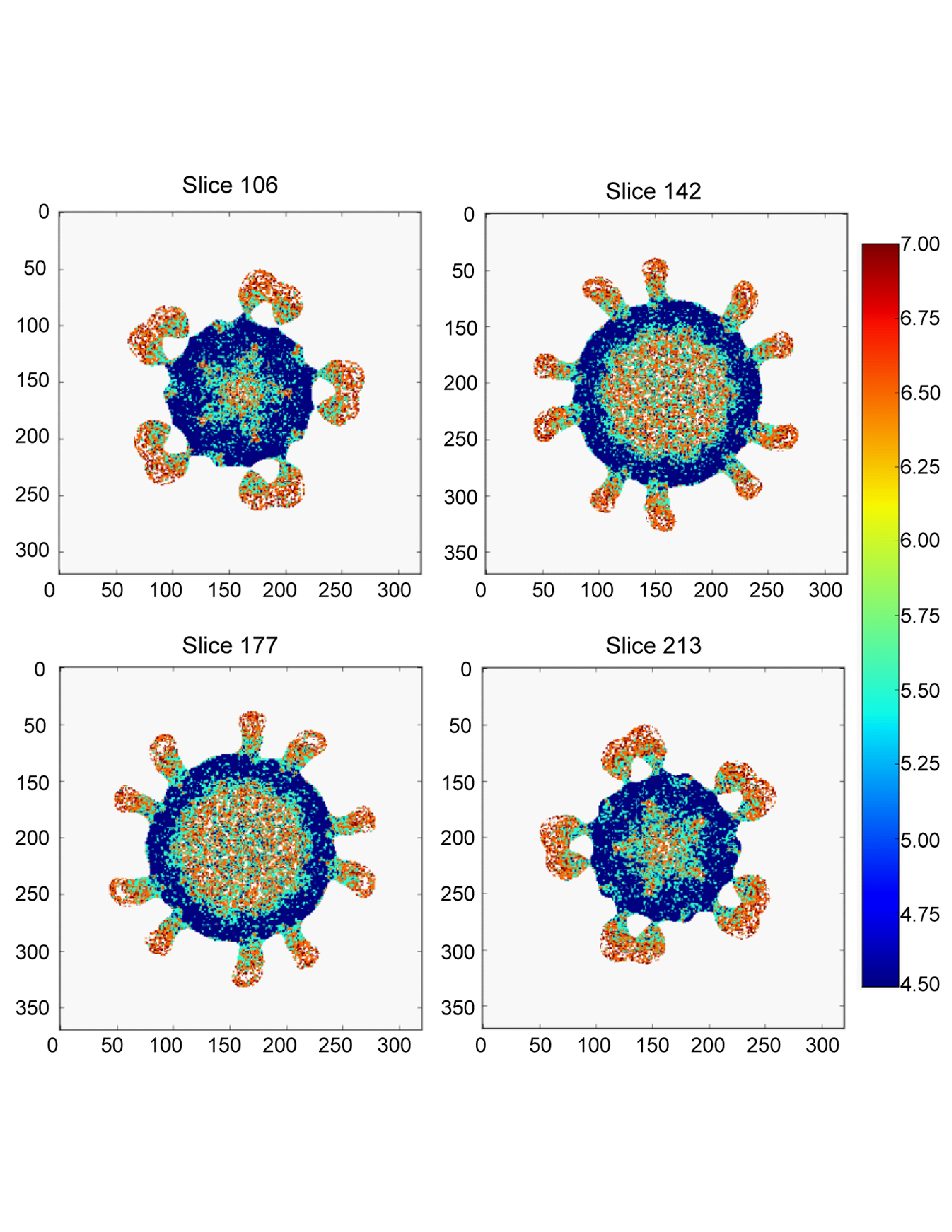

Supplement: S3 Fig — Local resolutions estimated by Resmap were rendered by four representative discrete 2D slides of the map. The color bar on the left labels the corresponding resolution (unit is Å), with the dark blue representing 4.5 Å and deep red representing 7.0 Å resolution. (TIF) [file ppat.1005454.s003.tif]

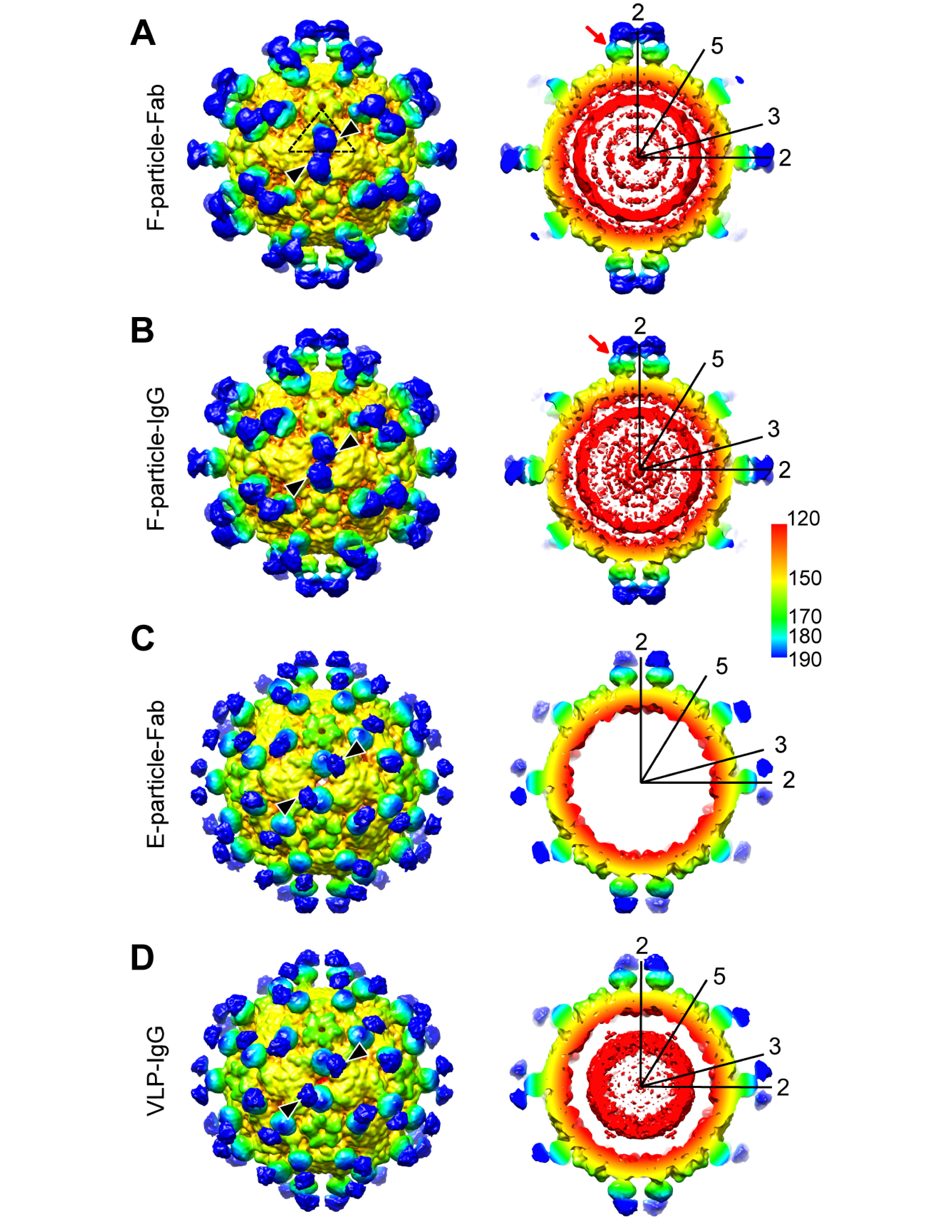

Supplement: S4 Fig — (A) F-particle-Fab complex. The complete particle is shown on the left. To better render the relative location of the bound Fabs/IgGs, the corresponding cut-away view of the central slice is also displayed on the right. (B) F-particle-IgG complex. (C) E-particle-Fab complex. (D) VLP-IgG complex. The same radial colour scheme from the centre of a sphere is used as in Fig 1A–1D. The icosahedral 5-fold, 3-fold and 2-fold symmetry axes are indicated in the cut-away view. The black arrow-heads on the left panels indicate a pair of adjacent Fab densities across the 2-fold axis, and the red arrows on the right panels indicate a hollow middle region between the two lobes in a Fab density. (TIF) [file ppat.1005454.s004.tif]

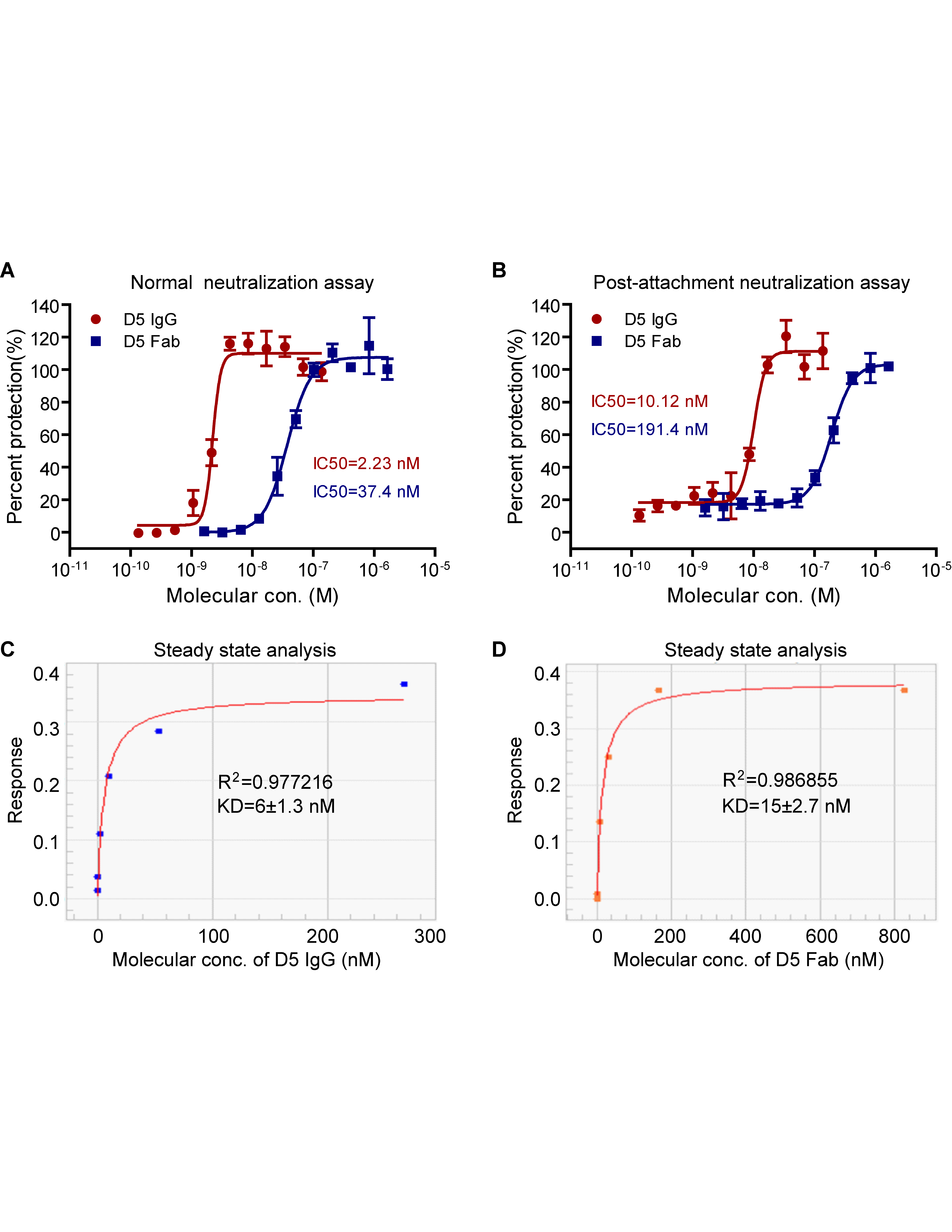

Supplement: S5 Fig — (A) IC50s determined by the standard neutralization assay. (B) IC50s determined by the post-attachment neutralization assay. The error bars indicate standard deviations of triplicate wells at each concentration. IC50s were calculated by GraphPad Prism 5.0. (C) Bio-layer interferometry analysis of D5 IgG. (D) Bio-layer interferometry analysis of D5 Fab. (TIF) [file ppat.1005454.s005.tif]

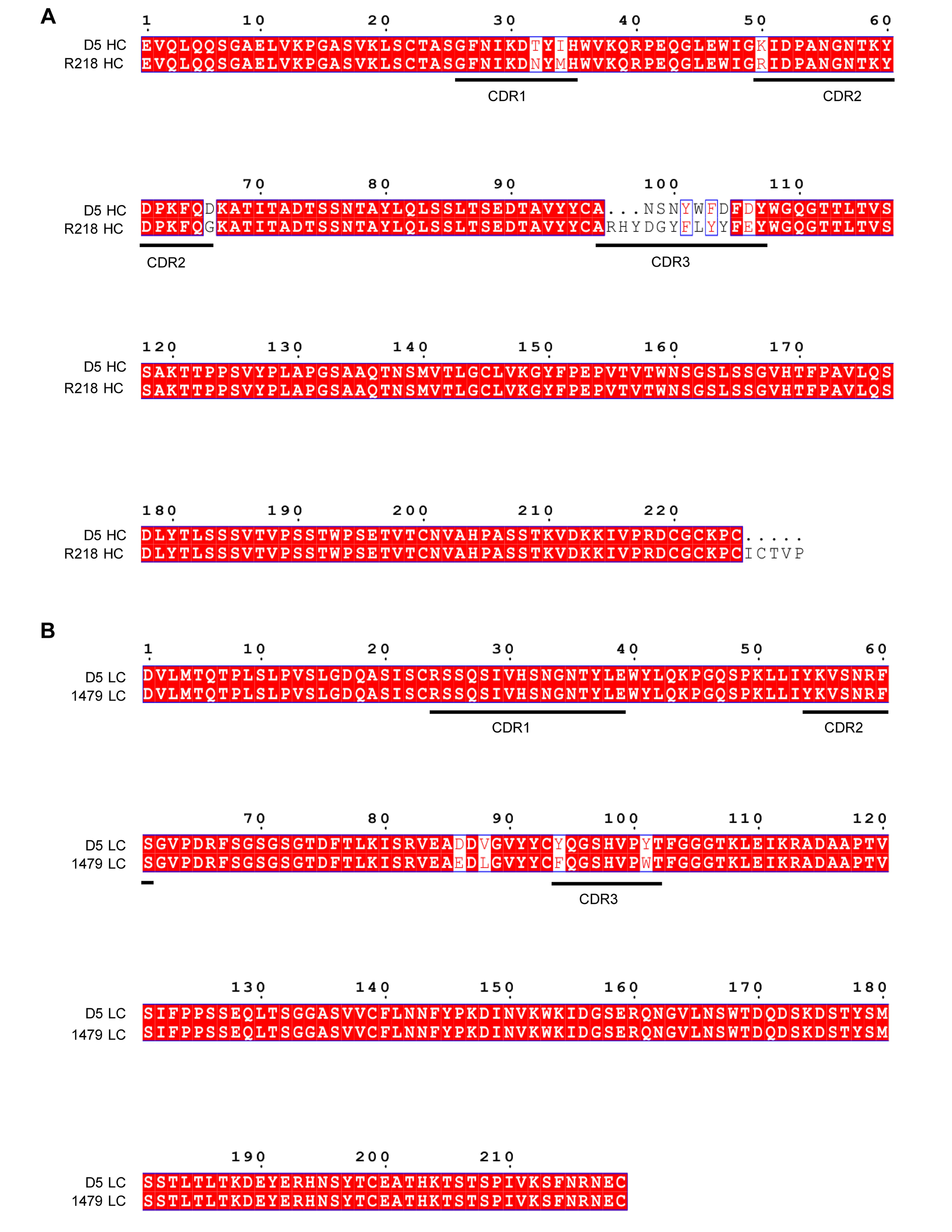

Supplement: S6 Fig — (A) In the variable region of heavy chain, antibody D5 shows high sequence identity with antibody K218 (PDB ID: 4K2U) in the CDR1 and CDR2 regions, but not in the CDR3 region. (B) In the variable region of light chain, D5 shows high sequence identity with antibody 1479 (PDB ID: 3U9U) in not only the CDR1 and CDR2 regions, but also the CDR3 region. (TIF) [file ppat.1005454.s006.tif]
