# Supplementary material for: Structural Basis for Recognition of Human Enterovirus 71 by a Bivalent Broadly Neutralizing Monoclonal Antibody
Source: PLoS Pathog. 2016 Mar 3;12(3):e1005454. doi: 10.1371/journal.ppat.1005454 (PMC4777393; doi:10.1371/journal.ppat.1005454)
Supplement: S1 Table — (DOCX) [file ppat.1005454.s007.docx]

**S1 Table.** Number of images and particles, and final resolution for the cryo-EM reconstructions.

| Structures | No. of images / particles | Final resolution |
| --- | --- | --- |
| F-particle-Fab | 320 / 2902 | 4.8 Å |
| F-particle-IgG | 81 / 244 | 7.2 Å |
| E-particle-Fab | 320 / 1303 | 6 Å |
| VLP-IgG | 192 / 2241 | 5.5 Å |
