# Supplementary material for: Structural Basis for Recognition of Human Enterovirus 71 by a Bivalent Broadly Neutralizing Monoclonal Antibody
Source: PLoS Pathog. 2016 Mar 3;12(3):e1005454. doi: 10.1371/journal.ppat.1005454 (PMC4777393; doi:10.1371/journal.ppat.1005454)
Supplement: S2 Table — * For the correlation calculation, the entire density maps were used (including the IgG/Fab). (DOCX) [file ppat.1005454.s008.docx]

**S2 Table.** Correlation scores between cryo-EM density maps* of different immune complexes, calculated by the Chimera *Fit In Map* module**.**

| Structures | F-particle-Fab | F-Particle-IgG | E-particle-Fab | VLP-IgG |
| --- | --- | --- | --- | --- |
| F-particle-Fab | -- | 0.981 | 0.788 | 0.772 |
| F-particle-IgG | 0.981 | -- | 0.744 | 0.728 |
| E-particle-Fab | 0.788 | 0.744 | -- | 0.984 |
| VLP-IgG | 0.772 | 0.728 | 0.984 | -- |

* For the correlation calculation, the entire density maps were used (including the IgG/Fab).
